# Phyco-synthesis of silver nanoparticles by environmentally safe approach and their applications

**DOI:** 10.1038/s41598-024-60195-3

**Published:** 2024-04-26

**Authors:** Sunita Choudhary, Geetanjali Kumawat, Manisha Khandelwal, Rama Kanwar Khangarot, Vinod Saharan, Subhasha Nigam, Harish

**Affiliations:** 1 Department of Botany, Mohanlal Sukhadia University, Udaipur, 313001 India; 2 Department of Chemistry, Mohanlal Sukhadia University, Udaipur, 313001 India; 3 Department of Molecular Biology and Biotechnology, Rajasthan College of Agriculture, Maharana Pratap University of Agriculture and Technology, Udaipur, 313001 Rajasthan India; 4 Amity Institute of Biotechnology, Amity University, Noida, 201313 Uttar Pradesh India

**Keywords:** *Asterarcys*, Silver nanoparticles, Antibacterial activity, Antifungal activity, Photocatalytic activity, Methylene blue, Microbiology, Pathogenesis, Nanoscience and technology

## Abstract

In recent years, there has been an increasing interest in the green synthesis of metallic nanoparticles, mostly because of the evident limitations associated with chemical and physical methods. Green synthesis, commonly referred to as "biogenic synthesis," is seen as an alternative approach to produce AgNPs (silver nanoparticles). The current work focuses on the use of *Asterarcys* sp. (microalga) for biological reduction of AgNO_3_ to produce AgNPs. The optimal parameters for the reduction of AgNPs were determined as molarity of 3 mM for AgNO_3_ and an incubation duration of 24 h at pH 9, using a 20:80 ratio of algal extract to AgNO_3_. The biosynthesized *Ast*-AgNPs were characterised using ultraviolet–visible spectroscopy (UV–Vis), zeta potential, scanning electron microscopy (SEM), Fourier-transform infrared spectroscopy (FTIR), X-ray diffraction (XRD), energy-dispersive X-ray spectroscopy (EDX), and high-resolution transmission electron microscopy (HR-TEM) with selected area electron diffraction (SAED) patterns. The nanoparticles exhibited their highest absorption in the UV–visible spectra at 425 nm. The X-ray diffraction (XRD) investigation indicated the presence of characteristic peaks at certain angles: 38.30° (1 1 1), 44.40° (2 0 0), 64.64° (2 2 0), and 77.59° (3 1 1) according to the JCPDS file No. 04-0783. Based on SEM and TEM, the *Ast*-AgNPs had an average size of 35 nm and 52 nm, respectively. The zeta potential was determined to be − 20.8 mV, indicating their stability. The highest antibacterial effectiveness is shown against *Staphylococcus aureus*, with a zone of inhibition of 25.66 ± 1.52 mm at 250 μL/mL conc. of *Ast*-AgNPs. Likewise, *Ast*-AgNPs significantly suppressed the growth of *Fusarium* sp. and *Curvularia* sp. by 78.22% and 85.05%, respectively, at 150 μL/mL conc. of *Ast*-AgNPs. In addition, the *Ast*-AgNPs exhibited significant photocatalytic activity in degrading methylene blue (MB), achieving an 88.59% degradation in 120 min, revealing multiple downstream applications of *Ast*-AgNPs.

## Introduction

New vistas in fundamental and applied nanotechnology are being opened up by the production and the utilisation of nanoscale matter for distinctive physicochemical and optoelectronic applications^1^. The two main types of nanoparticles (NPs) in the classification are organic (made from dendrimers, micelles, liposomes, and compact polymers) and inorganic (made from noble metals like silver, gold, copper, zinc, titanium, and palladium, etc.). Because of their high surface-to-volume ratio, they have the ability to drastically modify biological, chemical, and physical aspects^2^. Silver nanoparticles (AgNPs), which are among the metal nanoparticles, are particularly important in the domains of therapeutics and biology^3^. The antibacterial effects of AgNPs against many pathogens, such as bacteria, viruses, and fungus, are well established^4^. Nanoparticles (NPs) can be manufactured using physical, chemical, and green method^5^. Simple biosynthesised nanoparticles have recently been identified as significant nanomedicines for a wide range of biomedical uses, such as antimicrobial activity and cytotoxic action against different cancer cells. Among all the metal oxide nanoparticles, their composite has found application in pharmacology and medicine^6^**.** Sustainable (green) synthesis is an alternative to physical and chemical processes that utilise hazardous substances, surfactants and adverse conditions, such as high temperatures or excess energy. Green nanoparticle synthesis is relatively energy-efficient, sustainable, affordable, simple, and scalable for industrial production^7^. In order to sustain the value of green synthesised NP_S_, there are three prerequisites. (i) Opting for environmentally favourable systems of solvents (ii) A sustainable reducing agent and (iii) an appropriate capping agent for stabilising nanoparticles. A variety of fungus, plants, algae, and other microbes are utilised for green synthesis^8^. Seaweeds are the most promising source of bioactive metabolites and have been used in food products, wastewater remediation, and medicinal potential. An excellent source of bioactive metabolites with abundant secondary components that have a wide range of biological actions are seaweed-mediated nanoparticles. The biological activities of seaweeds, such as green, brown, and red algae, include antibacterial, antiviral, anthelminthic, spermicidal, anticoagulant, antioxidant, antithrombotic, immuno-inflammatory, and cytotoxic effects^6^**.** Among these algae are aquatic organisms that can perform photosynthetic reactions to fulfil their nutritional requirements^9^. Algal synthesis of AgNPs is especially intriguing due to algae's exceptional capacity to assimilate metals and reduce metal ions. These can be used as effective bioagents to remove heavy metal pollution because of their capacity to endure a wide range of harsh environmental conditions^10^. Algae is a plentiful and widely dispersed organism; their ability to thrive in a lab setting is an additional benefit. These organisms offer low-cost assistance in large-scale production. Cellular reductase, which results in a reduction during the synthesis of AgNPs, is the primary component that aids in the synthesis of AgNPs via algae. Researchers reported that algae can produce silver nanoparticles both intracellularly and extracellularly^11^. Proteins, lipids, carbohydrates, carotenoids, vitamins, and secondary metabolites are among the bioactive compounds found in green algae (Terpenoids, phenols, flavonoids, and alkaloids), stabilise the created nanoparticles by serving as both capping and reductant^7,8^. Numerous scientists have asserted the sustainable synthesis of AgNPs and their multiple uses using a variety of algal extracts, including *Sargassum myriocystum*^6^, *Chlorella pyrenoidosa*^12^, *Chlorella vulgaris*, *Chaetoceros calcitrans*^13^, *Scenedesmus abundans*^14^, *Caulerpa serrulate*^15^ etc.

Silver nanoparticles have numerous biological applications, including bio-imaging, bio-sensors, gene transport, photocatalysis, anti-microbial, anti-oxidant, and anti-cancer agents^7,16^. The antimicrobial activity of AgNPs is increased by interactions between Ag^+^ and bacterial cell walls, the inactivation of enzymes which linked to membranes, the assembly of bacterial cells, the impairment of vital bacterial biomolecules, the breakdown of the cell envelope, and the generation of Reactive Oxygen Species (ROS)^17^. AgNPs are employed by pharmaceutical firms because of their minimal toxicity to human cells and stability across a wide temperature range^18^.

Currently, the aquatic ecosystem is exposed to a significant amount of industrial effluent, involving hazardous dyes from the textile, printing, and paper industries, which poses a significant threat to ecosystems. Resistant dyes in effluent can be dissolved or absorbed using AgNPs. When AgNPs absorb visible solar light, the outermost electron is promoted to an increased energy level during the breakdown of a dye. Furthermore, the radicals produced by the oxygen molecule and hydroxyl ion accepting the excited electron contribute to decomposition of dye molecules adsorbed on the surface of AgNP. By accepting an electron from the dye, the hole generated on the AgNP orbital facilitates the dye's continued degradation^9^. Recent research has focused on the investigation of photocatalytic breakdown utilising biological metallic NPs by numerous scientists^19^. The photocatalytic elimination of organic pollutants by algal silver nanoparticles has recently attracted the attention of researchers^20^.

In the present study, aqueous extract of *Asterarcys* (microalgae) was utilised for the biological reduction of AgNO_3_ for green synthesis of AgNPs after thoroughly optimized parameters. The *Asterarcys*-mediated synthesis of silver nanoparticle denoted as “*Ast-*AgNPs” were characterized by UV–Vis spectroscopy (Ultraviolet–visible spectroscopy), Fourier-Transform Infrared Spectroscopy (FTIR), X-ray diffraction (XRD), Scanning Electron Microscopy (SEM), Energy-dispersive X-ray (EDX), High-Resolution Transmission Electron Microscopy (HR-TEM), and Zeta potential. The synthesized *Ast-*AgNPs were examined for their effectiveness against gram positive and gram-negative bacteria as well as fungal strains. Additionally, the photocatalytic activities of *Ast*-AgNPs against methylene blue (MB), was also examined.

## Materials and methods

### Materials

The sample of algae was collected from Lake in Udaipur. The chemicals used in the BG-11 medium were obtained from Hi-Media and Sigma-Aldrich. The following items were also obtained like Agar–agar from Hi-Media, Antibiotics (Penicillin G, Chloramphenicol, and Streptomycin sulphate) from Hi-Media, Silver nitrate from Merck, Muller-Hinton Agar (MHA) from Hi-Media, Potato Dextrose Agar from Hi-Media, and Methyl blue (MB) from LOBA Chemie. The Microbial Research Laboratory, Department of Botany at Mohanlal Sukhadia University, Udaipur, Rajasthan, India, provided several strains for evaluating the antibacterial and antifungal properties. These strains include *Staphylococcus aureus*, *Bacillus subtilis*, *Proteus vulgaris*, *Klebsiella pneumoniae*, *Fusarium* sp., and *Curvularia* sp.

### Collection of samples

Microalgal samples were collected from many places with fresh water bodies, including Fatehsagar Lake, Pichola Lake and Kukhadashwar temple pond from the Udaipur Region of Rajasthan, India. Water samples with a noticeable algal component were collected in labelled sampling polybags and Teflon bottles, then subsequently transferred them to the lab for additional research.

### Isolation, purification and establishment of axenic culture

To separate single strain of algae from mixed cultures, BG-11 medium was added to the collected samples. The enriched algal cultures in BG-11 media were carefully examined to confirm the presence of several species^21^. Serial dilution was used to isolate a single algal strain from the collected sample. The image was then examined under a light microscope (Olympus CH20i), and then striking was performed on culture plates with 20 mL of the solidified Agar-BG-11 growth media at the laminar air flow clean bench. It took several iterations of this procedure to obtain only one strain of algae. The isolated strain was treated to a triple antibiotic solution technique in order to generate axenic culture^22^. After two to three weeks of incubation, the colonies emerged, and they were separated and put into liquid media. Algal species were cultured in growth chamber with the temperature of 25 °C under a white, cold fluorescent light with 16:8 h light:dark period at pH 7.4 of media for additional studies. Centrifugation process at 4000 rpm (20 min, 4 °C) was employed to harvest axenic microalgal cultures. To prevent contamination, the pelleted biomass was rinsed thrice with sterile water (deionized). The harvested wet biomass of algae was dried in oven (Yorco Scientific industries) at room temperature (RT) for 24 h^23^.

### Identification of isolated algae

Under a calibrated compound light microscope (Olympus CH20i) with 10 ×, 40 ×, and 100 × immersion lenses, the main morphological characteristics of the isolated algal strain were examined and digital photomicrographs of the specimens were taken. For molecular identification, the genomic DNA extraction method (CTAB) was used to characterize the algal strain, which was then followed by PCR, gel electrophoresis, and algal identification was done by using *rbcL* (Ribulose bisphosphate Carboxylase Large subunit) gene sequencing. In addition, that sequence was uploaded to the NCBI database to obtain an accession number.

### Algal extract preparation

The algal extract was prepared following slightly modification of previously published protocol^24^. The oven dried algal biomass of *Asterarcys* sp. was pulverized into a fine powder. The algal extract was made by combining dried algal biomass and 100 mL Deionized water (DI water) in a 150 mL beaker and incubating it at 60 °C (30 min). The extract was centrifuged at 5000 revolutions per minute (RPM) for 15 min. Following centrifugation, the filtrate (after passing through Whatman 1 filter paper (grade B59501, 125 mm) was employed in the biosynthesis of AgNPs as a reducing and capping agent. The purified algal extract was refrigerated at 4 °C for further use.

### AgNO_3_ solution preparation

By weighing AgNO_3_ (Silver nitrate) in a specific quantity according to molarity and dissolving it in DI water, different concentrations of AgNO_3_ solution were obtained. After being thoroughly dissolved, the mixture was then employed for additional experiments.

### Synthesis of *Ast*-AgNPs

#### Optimization of synthesis of *Ast*-AgNPs

Optimizing factors such pH, reaction mixture temperature, the ratio of biomass to metallic ions, reaction time, and precursor concentration is crucial. Individual parameters were optimized one at a time while the other parameters kept constant. Changing the morphology of the end product, various reaction settings can have a significant influence on the reduction procedure. The "one factor at a time" method and the factor based experimental design were utilized throughout the course of the experiment^25^.

##### Effect of weight of algal biomass

To investigate the optimal conditions for AgNPs production, algal extract was prepared using various amounts of dried algal biomass (1, 3 and 5 gm).

##### Effect of molarity of AgNO_3_

Algal extract was mixed with solutions containing varying amounts of AgNO_3_ (1, 2, 3, 4, and 5 mM). The mixture was kept at the ambient temperature. A spectrophotometer was used to measure the color change (Shimadzu UV-1900).

##### Effect of algal extract and AgNO_3_ ratio

Algal extract and AgNO_3_ solution were added in five different ratios (5:95, 10:90, 15:85, 20:80, and 25:75). The reaction solution was allowed to incubate for 24 h at the ambient temperature. The peak of UV–Vis absorbance was examined in order to detect the synthesis of nanoparticles.

##### Effect of pH

Algal extract's pH could be adjusted to look at how pH affects the production of silver nanoparticles. Analytically graded 0.1 N NaOH and 0.1 N HCl standard solutions were added dropwise to adjust the pH of algal extract, within the range of 7, 8, 9, 10, and 11. The 20 mL of algal extract was added to 80 mL of 3 mM AgNO_3_, and the reaction solution was left for 24 h at ambient temperature. Utilising a UV–vis spectrophotometry with a visible wavelength (300–700 nm), the pH effect on the bio-fabrication process was determined.

##### Effect of temperature

The 80 mL of a 3 mM AgNO_3_ solution was combined with 20 mL of algal extract before being incubated at room temperature (25 °C), 40, 50, and 60 °C for 24 h. By varying color, the absorbance spectra were produced.

##### Effect of incubation time

The 80 mL of AgNO_3_ solution and 20 mL of algal extract were combined at various intervals throughout the incubation period. At specific intervals of 3, 6, 12, 24, and 48 h was used to analyze color fluctuations using UV–vis spectrophotometer.

#### Phyco‑synthesis of *Ast*‑AgNPs after optimization

**Figure 1:**
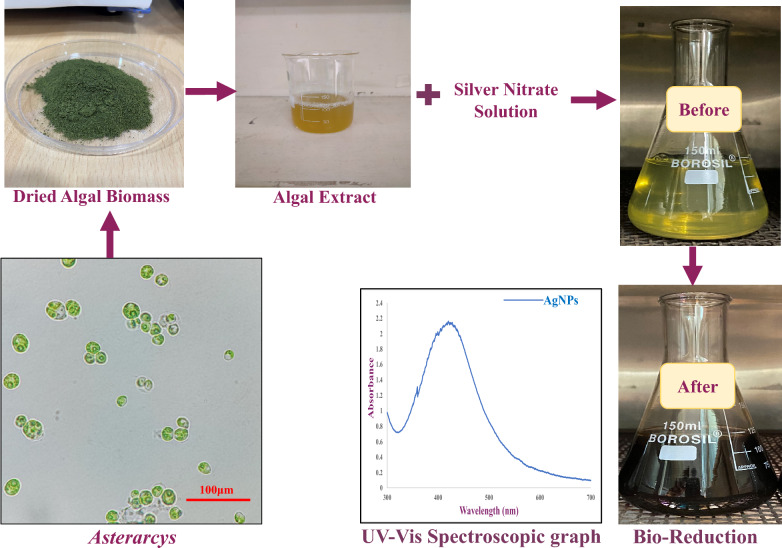
Schematic representation of green synthesis of Silver nanoparticles from *Asterarcys* extract.

In a 150 mL conical flask, 20 mL of algal extract at pH 9 was combined with 80 mL of 3 mM AgNO_3_ solution to produce silver nanoparticles. The mixture was subsequently kept for 24 h at ambient temperature^15^. After process was complete, the AgNPs were centrifuged (15,000 rpm) for 20 min at a temperature of 4 °C. The biogenic *Ast*‑AgNPs were repeatedly centrifuged with DI water to eliminate contaminants before being transferred to a beaker and lyophilized. For further investigations, dried particles were gathered and preserved (Fig. 1).

### Characterization of *Ast-*AgNPs

#### UV–visible spectra analysis (UV–Vis)

The phyco-synthesis of AgNPs is confirmed with the help of UV–vis spectrophotometer at wavelengths between 300 and 700 nm. AgNPs (0.50 mL) were taken in a quartz cuvette and 2.50 mL of deionized water were added to dilute them for the UV–Vis study. The reaction solution that was anticipated to produce nanoparticles was exposed to wavelengths from 400 to 500 nm in order to identify the suitable wavelengths for nanoparticles production^26^.

#### Scanning electron microscopy (SEM) analysis

The surface morphology of the fabricated AgNPs was examined by SEM (EVO 18, Zeiss, Germany). Just a tiny amount of the sample was used to produce the thin films on the carbon-coated gold grids. With the help of paper, excess solution was blotted off., and the grids were then dried under a mercury lamp for five minutes before the photos were taken^26^.

#### Energy-dispersive X-ray spectroscopy (EDX) analysis

EDX (EVO 18, Zeiss, Germany) carried out an elemental analysis of silver. The drop coating procedure was applied to prepare the sample of aqueous AgNPs suspension for the analysis of EDX. At several locations on the sample, EDAX analysis was done in the spot profile mode with a beam diameter of 1 µm.

#### High-resolution transmission electron microscopy (HR-TEM) analysis

The interior structure of AgNPs was examined using TEM (Tecnai, G-20 FEI, USA). Specimens were supplied in a copper frame and given a 30 min drying period and further images were captured.

#### Selected area electron diffraction (SAED) patterns analysis

Using the SAED approach, multiple lattice parameters were examined from a range of diffraction patterns in order to assess the crystalline structure of the nanoparticles^27^. Using a parallel stream of intense electrons, this method focuses attention on a small sample. The XRD results were verified by comparing the d-spacing of the circular rings in the diffraction patterns with the standard JCPDS databases^28^.

#### Zeta potential analysis

The NP’s negative electrostatic charges can reveal NP’s stability^29^. Using the Zeta-sizer Nano, (ZS90) Malvern, the consistency of the generated nanoparticles, which were colloid in nature, was subjected to zeta potential analysis to examine stability of synthesized AgNPs.

#### X-ray diffraction (XRD) analysis

The dried AgNPs were analysed through an X-ray diffractometer to identify their crystal structure. The XRD patterns were captured using CuK radiation with a wavelength of 1.5406 Å using a Rigaku Ultima IV X-ray diffractometer (Japan). The diffractometer was operated at 40 kV and 44 mA with a step size of 0.02° and a speed of scanning of 4°/min, scanning in the range of 2θ = 20°–80°. The crystalline nature of the nanomaterial has been determined by comparing the obtained results with the standard JCPDS database. The characteristic crystalline nature of nanomaterials has been determined using the Debye–Scherrer formula^30^.

$${\text{D }} = {\text{ K}}\lambda /\beta {\text{cos}}\theta$$

where D is the coherent scattering length (crystalline size in nm), K is the Scherrer's constant (0.98), λ is the wavelength of the X-ray source, β is the angular FWHM of the XRD diffraction peak and θ is the Bragg angle.

Using Origin Pro 2023 software, the FWHM was computed from the Gaussian function.

#### Fourier-transform infrared spectroscopy (FTIR) analysis

The functional groups of the algal constituents for the stabilisation of AgNPs and a reduction of Ag^+^ ions were identified using FTIR analysis. The *Ast*-AgNPs that had been biosynthesized were mixed with potassium bromide (Kbr) to create a pellet, which was subsequently tested for the presence of infrared spectral bands with a resolution of 4 cm and wavelengths between 4000 and 400 cm^−1^ using FTIR spectrophotometer (Bruker Alpha, USA)^26^.

### Applications of *Ast*‑AgNPs

#### Antibacterial activity

The bacterial strains like *Staphylococcus aureus*, *Bacillus subtilis*, *Klebsiella pneumoniae* and *Proteus vulgaris* were taken from the Microbial Research Laboratory at the Mohanlal Sukhadia University in Udaipur, Rajasthan, India. The obtained bacterial culture was sub cultured and placed in Muller-Hinton nutrient agar media (Hi-Media) in a Petri dish for further experiments.

##### Preparation of inoculums

To create bacterial inoculums, a loopful of bacterial culture was transferred from new agar plates to tubes containing 10 mL of Nutrient Broth (Hi-media), where it was cultured for 24 h at 37 °C. Occasionally, the culture tubes were shaken to aerate the contents and encourage growth^31^.

##### Procedure

Following Kathiraven et al. and Sinha et al. with a few minor modifications^24,32^, the anti-bacterial activity was performed on Muller-Hinton Agar (MHA) plates, the antibacterial efficacy of biologically produced silver nanoparticles against *Staphylococcus aureus, Bacillus subtilis, Proteus vulgaris*, and *Klebsiella pneumoniae* was examined by the agar well diffusion procedure. The sterilized MHA (25 mL/plate) was added to petri dishes and allowed for a while until it solidified (at least 15–20 min). Using a flame-sterile glass spreader, overnight broth cultures of each strain of microbes (100 μL) was evenly distributed on an MHA plate and given some time to fully absorb the inoculums. Each of these solidified MHA medium plates was hollowed out to a diameter of 6 mm using a sterilized cork borer. Using a micropipette, the following samples were added to each well of all the plates: different concentration of AgNPs, pure algal extract, AgNO_3_ solution, DI water (as a negative control), and streptomycin sulphate (as a positive control). The substances were then put in the wells and allowed to diffuse for 30 min at ambient temperature. Streptomycin, algal extract, AgNO_3_ and DI water are used at the same concentration (100 µL/mL), however silver nanoparticles are employed at varied concentrations (100–250 µL/mL). The petridishes were incubated for 24 h at 37 °C. A distinct inhibition zone created around each well after incubation, indicating antimicrobial activity. The zones of inhibition were assessed by taking measurements of the width of the inhibition zones and it proved that bacteria were being inhibited by nanoparticles. Three replicates of each experiment were carried out simultaneously.

#### Antifungal activity

The antifungal activity of *Ast*-AgNPs, against *Fusarium* sp. and *Curvularia* sp. were examined using the poison food method^33^. Silver nanoparticle stock solution (5 mg/mL) was prepared and placed at sonicator (Ningbo Sjialab Eqipment Co., Ltd) for 1 h homogenization process by probe-3. The 50 µL/mL, 100 µL/mL, and 150 µL/mL solutions were made from this stock solution. Additionally, a bavistin solution was made and used as a control. Algal extract was also employed in an antifungal assay to compare the extract's antifungal activity to that of synthesized *Ast-*AgNPs. The nine millilitres of sterilized PDA and 1 mL of AgNPs solution were mixed together and after that, the liquid was put into petri dishes and let to set for at least 15–20 min. Additional 6-mm diameter wells were created from 7-days old culture using a sterilized cork borer, put aseptically in the centre of solidified agar and maintained at 25 ± 2 °C in the incubator. After seven days, the average diameter of the growth was calculated. The % of mycelium growth inhibition used to measure the antifungal activity of each plate sample was calculated as follows^34^.

$${\text{Inhibition rate }}\% \, = \frac{{{\text{mycelial growth in control }} - {\text{ mycelial growth in treatment }} \times { 1}00}}{{\text{mycelial growth in control}}}$$

#### Photocatalytic activity

A number of oxidation reactions utilise AgNPs as a catalyst because of their high surface-to-volume ratio^35^. Methyl blue (MB) is from the member of the phenothiazine-family solid brown dye colourant with a molecular weight of 319.85 g/mole and the formula C_37_H_27_N_3_Na_2_O_9_S_3_. It is frequently employed as a colourant in numerous applications. Methylene blue, a cationic dye, was used to assessed the dye degradation potential of green synthesised AgNPs. The % of decolorization measured by the formula given^25^.

$$\% {\text{ Decolorization }} = \, \left( {{\text{C}}_{0} - {\text{C}}/{\text{C}}_{0} } \right)*{1}00$$

where, C_0_ = dye initial optical density, C = dye optical density after the photocatalytic decolorization.

##### Photocatalytic degradation of dye: operational parameters

The spectra at 664 nm demonstrate the photocatalytic breakdown of MB dye by AgNPs. Colour change is the indicator of Dye degradation^6^. The declining absorbance value, which occurs every 15 min, signals MB deterioration. The photocatalytic activity of *Ast*-AgNPs was examined in the presence of natural sunlight by adjusting the test solution's pH, initial dye concentration, catalyst concentration and reaction time^20^.

Effect of pH: The initial pH of the reaction solution affects the production of active radicals and the characteristics of the photocatalyst in the photocatalytic degradation process^36^. Studies were conducted utilising silver nanoparticle nano catalyst by calibrating the pH of the methylene blue dye solution to 3, 5, 7, 9, and 11 by adding standard solutions of 1N HCl or 1N NaOH, as pH plays important role in dye degradation.

Effect of MB dye concentration: The concentration of MB dye altered from 20 to 100 ppm while holding all other variables constant in order to determine the effect on photocatalytic activity (one factor at a time).

Effect of catalyst dosage: By shifting the catalyst concentration, the effect of catalyst dosage on MB dye degradation was investigated from 5 to 25 mg at an optimal pH of 11 and a dye concentration of 20 ppm.

Effect of light and catalyst: UV irradiation activated the catalyst, causing *OH radicals to appear on the surface^36^. We conducted three sets of reactions: (1) with the catalyst in the dark (2) with the catalyst in light (3) without the catalyst in light, in order to determine if the degradation happened through adsorption, photolysis or photocatalysis. In both the presence of the catalyst in the dark and the absence of the catalyst in the light, we found that there was barely any dye degradation. The catalyst and light were the conditions where dye degradation happened at the highest rate.

### Statistical analysis

The entire set of data were statistically analysed using GraphPad Prism (version 3.02) software. The statistically significant difference is calculated with a one-way ANOVA, and the p-value denotes the likelihood of error.

## Results and discussion

### Algal Identification

Microscopic images of isolated microalga demonstrated that the algal cells were non-motile and spherical in shape. Nonetheless, it was extremely challenging to properly categorise the isolate based solely on morphological characteristics. However, the rbcL sequence found to have the greatest degree of similarity to the alga *Asterarcys quadricellulare* (accession number MW560279).

### Synthesis of *Ast*-AgNPs

**Figure 2:**
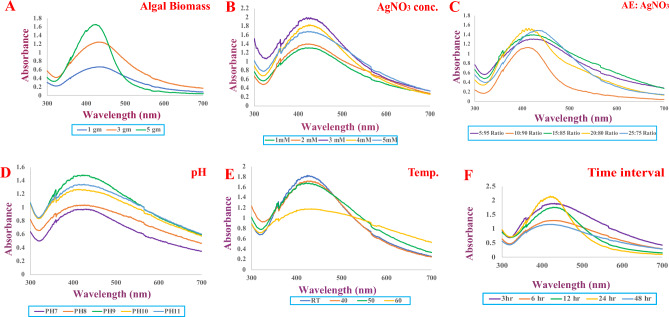
UV–Visible spectroscopic graphs of optimized parameters for AgNPs synthesis likewise, (**A**) algal biomass, (**B**) conc. of AgNO_3_, (**C**) AE:AgNO_3_, (**D**) pH, (**E**) temperature, (**F**) time interval.

The *Asterarcys* sp. algal extract was investigated as a possible substitute for harsh chemical reagents such sodium borohydride as a reducing, capping, and stabilizing agent. The AgNPs' sizes and morphologies are influenced by a variety of variables, including pH, AgNO_3_ and reducing agent concentrations, incubation duration, temperature and preparation techniques^37^. To generate NPs that are smaller and more stable, numerous variables, including metallic concentrations, the ratio of algal extract and AgNO_3_, temperature, pH, light, and duration of incubation, were investigated (Fig. 2A–F). In this study varied weights of dried algal biomass were used for optimisation to synthesize AgNPs. The findings showed that biomass had a significant influence on the formation of AgNPs. Algal extract with 5 gm of dried algal biomass resulted in a narrow band with a high intensity peak at 430 nm (Fig. 2A) suggesting this more appropriate conc. over 1 and 3 gm of algal dry wt. Optimising the AgNO_3_ molarity is essential for producing appropriate size of AgNPs needed for subsequent studies since it significantly affects particle size^38^. When the quantity of AgNO_3_ was rise-up from 1 to 3 mM, a large amount of AgNPs were generated, and a higher UV–Vis absorbance peak (428 nm) was observed (Fig. 2B). But from 3 to 5 mM of AgNO_3_, it revealed the lower peak. Thus, the best optimized concentration of AgNO_3_ solution was determined to be 3 mM. Similar outcomes were also observed when AgNPs were synthesised using a green technique^39^. *Asterarcys* extract concentrations (up to 20%) were added to a silver nitrate (AgNO_3_) solution, and it was found that this increased the absorbance band intensity. The red shift in the synthesised AgNPs is related to the increased concentration of algal extract and AgNO_3_ ratio (from 20 to 25%). The highest absorbance peak (428 nm) was achieved at a 20:80 (algal extract: silver nitrate) ratio (Fig. 2C). A comparable investigation on *Chlorella vulgaris* was also carried out by Rajkumar and associates. Similar results were seen when AgNPs were produced using the *Chlorella vulgaris* algal extract serves as a reducing and capping agents. The results showed that AgNPs were produced at the: 8:2 v/v extract ratio^40^. Both the colour of the reaction solution and the UV–Vis spectrum have been reported to be affected by change of the pH of the solution. The effects of different pH levels on the fabrication of AgNPs were investigated (Fig. 2D). The highest absorption peak was seen at 425 nm at pH 9 in this experiment. Small-sized nanoparticles were produced in an alkaline pH environment, whereas large-sized nanoparticles were produced in an acidic pH environment. Numerous functional groups are said to be ionised at an alkaline pH, making them available for reduction and facilitating the fabrication of NPs of small size^41^. The synthesis of AgNPs at various temperatures (RT °C, 40 °C, 50 °C, and 60 °C) is also investigated and results are shown in Fig. 2E. The silver nanoparticles synthesized at RT °C exhibited highest peak at 426 nm. The optimal temperature for generating NPs was determined to be ambient temperature. As the temperature changes, bands also become wider^40^. The reaction time when the silver nitrate reacts with the extract also determines the quantity of nanoparticles produced^42^. As reaction time increased, absorbance spectra at 425 nm increased, and colour intensity enhanced over the duration of the incubation period. The greatest absorption was seen 24 h after the incubation period (Fig. 2F). Dashora et al. also made similar observations regarding incubation time^33^. The UV–Vis spectra measurements revealed that the colour intensity increased over the course of the different time intervals up to 24 h, proving that AgNPs were synthesised without agglomeration. Comparable investigation has been carried out on the generation of AgNPs employing olive oil leaf extract^43^.

The best optimised conditions for the production of *Ast-*AgNPs were discovered to be as incubation temperature (RT °C), extract: silver nitrate ratio (20:80), ionic strength of extract (pH-9), AgNO_3_ molarity (3 mM), and maximum incubation length (24 h). The conversion of Ag^+^ ions into AgNPs by biomolecules in the supernatant was verified by the presence of a significant UV–Vis spectra peak at 425 nm (Fig. 1). Similarly, AgNPs produced by *Palmaria decipiens* exhibited an absorption maximum at 425 nm^44^. The silver nanoparticles mediated by *Sargassum myriocystum*, a marine algae, showed characteristics peak at 420 nm^6^. On the other hand, the UV–Vis peak for AgNPs produced by *Chlorella ellipsoidea* algal extract was detected at 436 nm^45^. Previous studies have already shown that various silver nanoparticles from microalgae exhibited a peak in the wavelength region of 410–450 nm^46^.

### Characterization of *Ast-*AgNPs

**Figure 3:**
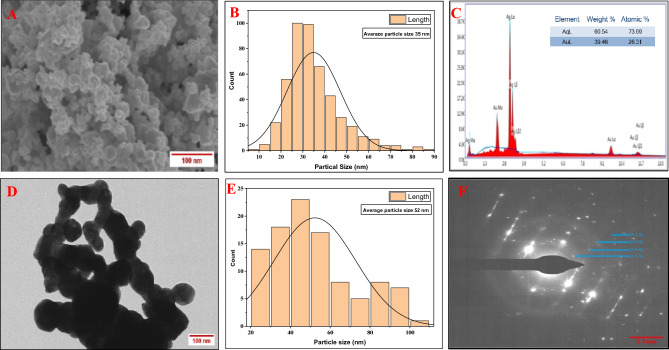
Structural characterization of *Ast*-AgNPs; (**A**) SEM micrograph (scale bar-100 nm), (**B**) SEM histogram, (**C**) EDX, (**D**) TEM micrograph (scale bar 100 nm), (**E**) TEM histogram, (**F**) SAED pattern (Scale bar-5 1/nm).

**Figure 4:**
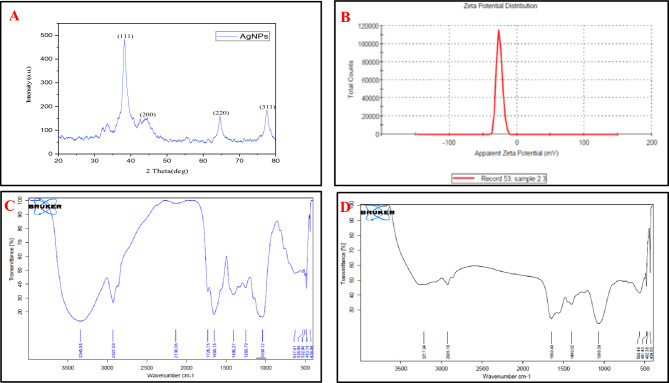
Characterization of *Ast*-AgNPs; (**A**) XRD, (**B**) zeta potential, (**C**,**D**) FTIR of Algal extract and AgNPs.

The size of *Ast*-AgNPs has been found to be within 10 and 90 nm, with an approximate mean size of 35 nm (Fig. 3A,B). These SEM findings were corroborated by previous published research^47^. The AgNPs with a spherical shape were found in *C. vulgaris* and *C. calcitrans*, with dimensions of 50–70 and 30–35 nm, respectively. The EDX observation that *Ast-*AgNPs contained 60.54 weight percent of silver implies a pure synthesis of silver nanoparticle (Fig. 3C). The nanoparticle’s EDX analysis revealed that silver (Ag^+^) atoms accounted for the greatest percentage of the peak intensity^13^. The EDX investigation for silver particles revealed a high signal in between 2.8 and 3.4 keV region^48^. The TEM micrograph (Fig. 3D) of the AgNPs revealing that *Ast-*AgNPs are polyform, quasi spherical, rectangular and triangular in shapes. The 52 nm was found to be the average size of nanoparticles ranging in size from 20 to 100 nm (Fig. 3E). The shape of the nanoparticles was described by other researchers as spherical, hexagonal and of a moderately wide range of sizes^46^. The crystal structure of NPs supported by selected area emission diffraction (Fig. 3F) The SAED pattern generated the interplanar d-spacings of 2.29, 2.10, 1.45, and 1.23. The diffraction rings with the characteristic patterns were indexed (1 1 1), (2 0 0), (2 2 0), and (3 1 1) and are compatible with the FCC (Face-centered cubic) lattice structure often seen in AgNPs Joint Committee on Powder Diffraction Standards (JCPDS) file no. 00-004-0783. Result are in parity with previous reports^49^. The XRD pattern of synthesised AgNPs was analysed and compared to the standard powder diffraction card designed by the JCPDS. According to the FCC structure of AgNPs, intense diffraction peaks attributable to AgNPs are plainly observed at 38.30, 44.40, 64.64, and 77.59, which correspond to the (1 11), (2 0 0), (2 2 0), and (3 1 1) Bragg's reflection planes (Fig. 4A)^32^. It was noted that reflections were sharp and intense, demonstrating the highly crystalline character of the produced AgNPs. When leaf extracts from *Ficus virens* were used to facilitate the synthesis of AgNPs, comparable outcomes have been found^50^. The synthesised nanoparticle’s Zeta potential values were determined to be -20.8 mV with a single peak (Fig. 4B), suggesting moderately stable dispersion in the solution. The negative potential value of biosynthesized AgNPs indicates the existence of bio-organic constituents serving as a capping agent in the extract^48^. Due to the negative value, synthesised *Ast*-AgNPs did not aggregate and remained in suspension^33^. Understanding the functional groups involved in the interactions between metal particles and biomolecules is made possible with the use of FTIR analysis. In this investigation, the *Asterarcys* sp*.* algal extract that stabilises and caps the AgNPs was identified using FTIR spectrum. In order to establish the functional groups, the measured intensity bands were assessed against standard values absorption bands in the FTIR spectrum at 3217, 2926, 1650, 1400, 1068, and 563 cm^−1^ indicate the presence of a capping agent on the NPs (Fig. 4C,D). Bands in the spectrum at 3217 cm^−1^ correspond to O–H stretching vibrations, indicating the presence of phenol and alcohol. The C–H stretching of the aromatic compound was observed to produce bands at 2926 cm^−1^. The 1650 cm^−1^ spectral band, which corresponds to C–N and C–C stretching, indicates the presence of proteins. The band at 1400 cm^−1^ is attributed to the N–H stretch vibration in protein amide bonds. Numerous studies indicate that these functional groups contribute to the stability and capping of AgNPs. The bands at 1068 cm^−1^ correspond to the protein's C–N (amines) stretch vibration. Alkyl halide’s typical C–Br stretching may be responsible for the band at 563 cm^−1^ area. The FTIR spectroscopic analysis may lead to the conclusion that interactions between proteins and Ag^+^ ions or nanoparticles in the algal extract have no impact on the proteins' secondary structure. In a related work, silver nanoparticles made from Urtica dioica leaves were also reported^51^. The wavelength absorption bands observed and presented as well as the FTIR spectrum of the algal extract and *Ast*-AgNPs, correspond with prior research^52^.

### Applications of *Ast-*AgNPs

#### Antibacterial studies

**Figure 5:**
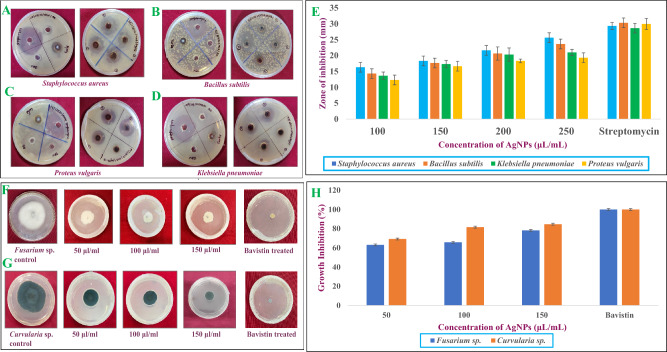
Anti-bacterial activity of *Ast*-AgNPs against different bacterial strains (**A**) *Staphylococcus aureus* (**B**) *Bacillus subtilis* (**C**) *Proteus vulgaris* and (**D**) *Klebsiella pneumoniae* in both control and treatment conditions by agar well diffusion method, (**E**) Graphical representation of zone of inhibition of all four bacterial strain against different concentration of AGNPs. Anti-fungal activity of *Ast*-AgNPs against different fungal strains (**F**) *Fusarium* and (**G**) *Curvularia*, (**H**) Graphical representation of percentage growth inhibition of two fungal strains against different concentration of AgNPs.

In the present study, the antibacterial activity of phyco-fabricated *Ast*-AgNPs was investigated against *Staphylococcus aureus* (gram + ve) (Fig. 5A), *Bacillus subtilis* (gram + ve) (Fig. 5B), *Proteus vulgaris* (gram-ve) (Fig. 5C), and *Klebsiella pneumoniae* (gram-ve) (Fig. 5D), with the results demonstrated that maximum antibacterial activity is reported against *Staphylococcus aureus* with a zone of inhibition as 25.66 ± 1.52 mm at 250 μL/mL, and lowest antibacterial activity is reported against *Proteus vulgaris*, measured at 19.33 ± 1.52 mm at 250 μL/mL (Fig. 5E). This could be explained by the perception that the NPs enter within the bacterium and cling to the cell membrane, preferably, target the respiratory chain, where they cause cell division and ultimately apoptosis. In the bacterial cells, the NPs cause the release of silver ions, which improves the bactericidal activity. Multiple studies suggest that AgNPs may adhere to the plasma membrane's surface and interfere with the cell's permeability and respiration processes^32^.

#### Antifungal activity

In this study, different concentrations (50, 100, and 150 μL/mL) of *Ast*-AgNPs against pathogenic fungi were tested for their antifungal efficiency. Remarkable antifungal activity was shown by *Ast-*AgNPs against fungi *Fusarium* sp. and *Curvularia* sp (Fig. 5F,G). *Curvularia* sp showed the highest percentage growth suppression by *Ast-*AgNPs at 150 μL/mL, at a rate of 85.05%, followed by 81.62% at 100 μL/mL and 69.66% at 50 μL/mL (Fig. 5H). The results demonstrates that as silver nanoparticle concentration was increased, growth inhibitory percentage increased as well. At 150 μL/mL, the AgNPs had a 78.22% effectiveness rate against *Fusarium* sp. growth inhibition, followed by 65.78% at 100 μL/mL and 63.12% at 50 μL/mL (Fig. 5H). The antifungal activity is caused by Ag^+^, leading to in membrane depolarization, pits in the cell wall, and the creation of plasma membrane apertures, thereby disrupting the integrity of the fungal membrane and obstructing the fungal cell cycle^53^. The algal extract of *Asterarcys* sp. lacked antibacterial properties. The extract exhibited minimal or no susceptibility in microbial strains, but the green synthesised AgNPs shown potent antibacterial activity against the pathogens^54^.

#### Photocatalytic activity

Phyco-synthesized AgNPs were used to investigate the photocatalytic dye degradation of methylene blue. The early indication that the dye was degrading came from a change in colour in the dye solution. In the meanwhile, AgNP concentrations, MB dye, and time intervals were found to influence photocatalytic activity. Silver nanoparticle plays a potential role in photocatalytic activity. Due to the SPR action of synthesised AgNPs, solar radiation transports e- from the valence band to the conduction band. The *O^−2^ is created when the excited electrons interact with O_2_ species. O_2_ + 2H ++ 2e− → H_2_O_2_ is formed as a result of the reaction between *O^−2^ and H+. All varieties of cationic dye can be degraded by the *OH radicals produced when H_2_O_2_ and H+ react^55^.

##### Effect of pH

**Figure 6:**
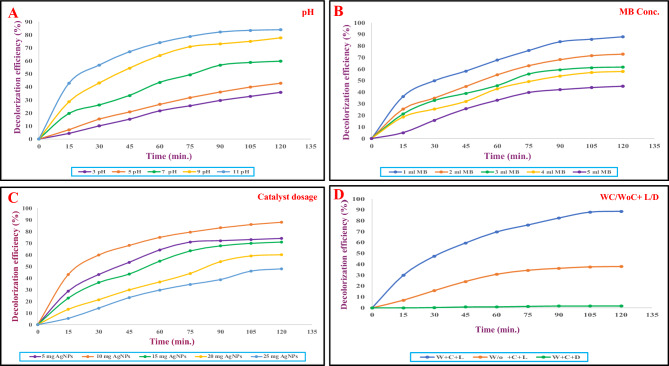
Optimized parameters for MB degradation; (**A**) pH, (**B**) MB Conc., (**C**) catalyst dosage, (**D**) WC (with catalyst); W/o (without catalyst), L (Light), D (Dark).

The initial pH of the reaction solution affects the generation of active species (radicals) and the characteristics of the photocatalyst in the photocatalytic system^56^. The results demonstrated in Fig. 6A showed that the solution pH had a substantial impact on the photodegradation rate of MB dye, with 11.0 being the ideal pH. It is clear that lowering the MB working solution’s pH caused the decrease in decolorization efficiency of *Ast-*AgNPs from 59.78% and 42.95%, at neutral pH and 5 pH, respectively. Contrarily, as the MB solution's alkalinity rose, its decolorization effectiveness increased, reaching 77.67% and 83.9% at respective pH levels of 9 and 11. The results demonstrated that the photocatalytic efficiency increased as the pH increased. With optimum degradation at pH 11, alkaline conditions were found to promote better photocatalytic degradation. The alkaline pH of the MB solution enhanced the generation of OH*, thus speeding up the reaction rate and enhancing the solution's degradation efficiency. Several researchers used AgNPs under UV/solar light to degrade rhodamine B as well as MB and observed comparable outcomes^36^.

##### Effect of MB dye concentration

It was found that the 20 ppm dye concentration show maximum 87.92% of degradation at 120 min. Figure 6B displayed the final results of effect of dye concentration on catalytic activity. On increasing the dye concentration of MB, the percentage degradation drastically decreased and reached 45.19% at 120 min (100 ppm). As a result, 20 ppm of dye concentration was found to be optimal. The efficiency of *Ast* -AgNPs' decolorization reduced when the original dye concentration was increased significantly. It is widely accepted that MB decolorization is sensitive to light process. Multiple-layer adsorption of dye molecules across the catalytic surface reduces UV light's ability to promote photo-oxidation as dye concentration increases. This would result in an abrupt decrease in the number of OH* assaulting MB molecules, thereby reducing the decolorization efficiency. It suggests that in order to have a same efficacy, the quantity of hydroxyl free radicals should rise proportionally to MB concentration. However, the photocatalytic degradation efficiency decreased after reaching the optimal initial dye concentration level^57^.

##### Effect of catalyst dosage

By adjusting the catalyst loading of *Ast-*AgNPs from 5 to 25 mg at an optimised 11 pH and a dye concentration of 20 ppm, catalyst dosage effect on the degradation % of MB dye was examined. It was noted that after 120 min, the 10 mg dosage of the catalyst showed a maximum 87.96% of degradation (Fig. 6C). However, after the optimum dose (10 mg), the reaction mixture have turned turbid due to an increase in the amount of *Ast-*AgNPs that blocked UV light, which inhibited photochemical activation and produced OH radicals^36^. This occurred when numerous light backscattering at the fluid's interface appeared to reduce overall light absorption, preventing light from penetrating deeply into a solution with a high concentration of NPs^58^. The effect of light exposure duration on decolorization effectiveness was investigated for a maximum of 120 min.

##### Optimized protocol for MB dye decolorization

The purpose of the optimisation was to address the highest starting MB concentration while minimising irradiation duration at a pH and catalyst concentration that is practically applicable. To establish a optimized protocol for MB dye decolorization, these four crucial conditions were taken into consideration. Under the experimental conditions specified, the maximum amount of MB decolorization (88.59%) was anticipated for a photocatalytic mechanism in which an initial MB concentration of 20 ppm was subjected to treatment with *Ast*-AgNPs at 10 mg concentration, calibrated to 11 pH, and exposed to radiation for an overall exposure time of approximately 120 min (Fig. 6D). The distinctive MB dye absorbance peak (664 nm) was observed dropping continuously with irradiation time, indicating that the dye was eliminated either by adsorption on the surface of the AgNPs or by catalytic degradation^20^. When it comes to the photodegradation of MB, light is crucial. With an increase in light exposure time, the photodegradation activity of AgNPs, TiO_2_, and Ag/TiO_2_ nanomaterials increases. It is so because the photoelectron is produced when visible light is used to excite the valence electron. OH * generated by these highly powerful photoelectrons cause photodegradation of MB^57^.

## Conclusion

The current study is significant since first time isolated microalga *Asterarcys* sp. was utilized in the biological reduction of AgNPs in a safer and more environmentally friendly manner. The AgNO_3_ molarity (3 mM) with maximum incubation period of 24 h at pH 9 were observed to be the best optimized parameters for the AgNPs synthesis. AgNPs was determined with an estimated mean size of 35 nm and 52 nm by SEM and TEM, respectively. The EDX analysis confirmed 60.54 of Ag in AgNPs. Zeta potential value for the synthesized nanoparticle were found to be − 20.8 mV with a single peak suggesting that synthesised *Ast*-AgNPs did not aggregate and remained suspended. Utilising FTIR spectra, the biomolecular functional groups required for the bio reduction of Ag^+^ and the capping/stabilization of AgNPs were identified. *Staphylococcus aureus*, *Bacillus subtilis*, *Klebsiella pneumoniae*, and *Proteus vulgaris* were among the Gram positive and Gram-negative bacteria that were significantly inhibited by *Ast*-AgNPs. In terms of antibacterial activity, *Staphylococcus aureus* had the highest levels of ZOI. An antifungal assay for biosynthesized nanoparticles was conducted utilising the poison food approach on *Fusarium* sp. and *Curvularia* sp. *Curvularia* sp. showed the highest growth inhibition percentage in antifungal activity. Furthermore, it was demonstrated that AgNPs have the ability to photocatalytically degrade hazardous dye methylene blue, in an aqueous solution. Methylene blue (MB) was potentially photocatalyzed by the *Ast*-AgNPs, with 88.59% of it being degraded in 120 min (Supplementary Information S1).

### Supplementary Information

Supplementary Information.

## Data Availability

The datasets generated during and/or analysed during the current study are available from the corresponding author on reasonable request.
