# Supplementary material for: Phyco-synthesis of silver nanoparticles by environmentally safe approach and their applications
Source: Sci Rep. 2024 Apr 26;14:9568. doi: 10.1038/s41598-024-60195-3 (PMC11053078; doi:10.1038/s41598-024-60195-3)
Supplement: Supplementary file 1 — Supplementary Information. [file 41598_2024_60195_MOESM1_ESM.pdf]

Phyco-synthesis of silver nanoparticles by environmentally safe approach and their applications

Sunita Choudhary[1], Geetanjali Kumawat[1], Manisha Khandelwal[2], Rama Kanwar Khangarot[2], Vinod Saharan[3], Subhasha Nigam[4], Harish*[1]

1. Department of Botany, Mohanlal Sukhadia University, Udaipur, 313001.
2. Department of Chemistry, Mohanlal Sukhadia University, Udaipur, 313001.
3. Department of Molecular Biology and Biotechnology, Rajasthan College of Agriculture, Maharana Pratap University of Agriculture and Technology, Udaipur 313001, Rajasthan, India.
4. Amity Institute of Biotechnology, Amity University, Noida, 201313, Uttar Pradesh, India.

*Corresponding author: Harish

Email: harish.botany1979@gmail.com

harish@mlsu.ac.in

Mobile: +91-94144-78466

Title: Phyco-synthesis of silver nanoparticles by environmentally safe approach and their applications

Supplementary table 1. SAED patterns for Interplanar d-spacing and corresponding lattice planes of *Ast-AgNPs*

**S.No.**	**1/2r (nm^{-1})**	**1/r (nm^{-1})**	**r (nm)**	**d-spacing(Å)**	**h k l**
1.	8.71	4.36	0.23	2.30	1 1 1
2.	9.48	4.74	0.21	2.11	2 0 0
3.	13.72	6.86	0.15	1.46	2 2 0
4.	16.14	8.07	0.12	1.24	3 1 1

Supplementary table:2 XRD Analysis of *Ast-AgNPs* with JCPDS, file No. 04-0783

**S. No.**	**2 θ**	**FWHM ((Full width at half maximum)**	**h k l (miller indices)**
1.	38.31	1.25	1 1 1
2.	44.41	2.90	2 0 0
3.	64.64	1.27	2 2 0
4.	77.60	1.48	3 1 1

Supplementary table 3: Functional group analysis of *Ast-AgNPs* by FTIR

**Sample**	**Absorption(cm^{-}1)**	**Bond/stretching**	**Functional groups**
Extract of *Asterarcys sp.*			
	3340.88	N–H stretch, O–H stretch, H-bonded	1°,2° amines, amides, carboxyl, phenols
	2136.06	–C≡C– stretch	alkynes
	1650.16	N–H bend	1° amines
	1406.27	N–O symmetric stretch	Nitro compounds
	1039.12	C–N stretch	aliphatic amines
Ast-AgNPs	3217.94	O–H stretch, H bonded	Alcohols, phenols
	2926.18	C-H stretch	alkanes
	1650	N–H bend	1° amines
	1400.62	–NO2 (aliphatic) or C–H (plane bend)	nitro groups or alkenes
	1068.09	C–N stretch	aliphatic amines

Supplementary table 4: Zone of Inhibition (mm) of *Ast-AgNPs* against bacterial strains *(Staphylococcus aureus*, *Bacillus subtilis*, *Klebsiella pneumoniae*, *Proteus vulgaris*) by agar-well diffusion method (Temp.: 37°C, Time: 24hrs)

**Bacteria**	**Zone of inhibition (mm diameter) AgNPs**				**Streptomycin Sulphate** 100μL/ml
	100μL/ml	150μL/ml	200μL/ml	250μL/ml	
Staphylococcus aureus	16.33±1.52	18.33±1.52	21.66±1.52	25.66±1.52	29.33±1.15
Bacillus subtilis	14.33±1.52	17.66±1.52	20.66±2.08	23.66±1.52	30.33±1.52
Klebsiella pneumoniae	13.66±1.15	17.33±1.15	20.33±2.08	21.00±1.11	28.66±1.52
Proteus vulgaris	12.33±1.52	16.66±1.52	18.33±0.57	19.33±1.52	30.00±1.73

Supplementary table 5: Growth Inhibition (%) of *Ast-AgNPs* against fungal strains (*Fusarium* and *Curvularia*) by poison food method (Temp.: 25±2°C, Time: 7days)

**Growth Inhibition (mm) Control**	**Name of Fungi**	**Growth Inhibition (%)**		
		**50μL/mL**	**100μL/mL**	**150μL/mL**
75	*Fusarium* sp.	63.12	65.78	78.22
78	*Curvularia* sp.	69.66	81.62	85.05

Supplementary table 6: Photocatalytic activity of *Ast-AgNPs* against MB**.**

**Time**	***WC+L**	***W/oC+L**	***WC+D**
0	0	0	0
15	29.92	7.01	0.01
30	47.48	15.91	0.26
45	59.52	24.24	0.86
60	69.71	30.87	0.96
75	76.02	34.52	1.46
90	82.5	36.32	1.78
105	87.9	37.64	1.81
120	88.59	37.98	1.89

Supplementary table 7: Optimized reaction conditions for the degradation of MB dye by *Ast-AgNPs*

**S. No.**	**Optimized parameters**	**Optimized values**
1.	pH	11
2.	Concentration of dye	20 ppm
3.	Catalyst dose	10mg

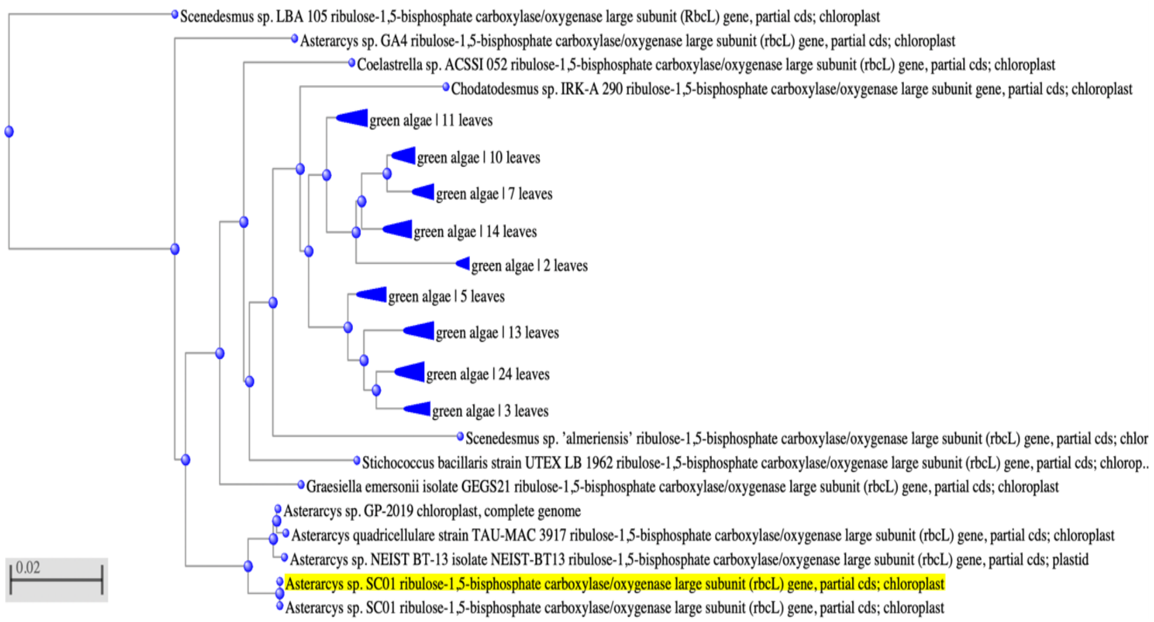

Supplementary figure 1: Phylogenetic tree of unicellular microalga *Asterarcys* MW560279 using *rbc*L gene sequence (partial CDS). Tree was constructed by NCBI Blast program.
